# Clinical presentation and prognostic analysis of adult patients with Langerhans cell histiocytosis with pulmonary involvement

**DOI:** 10.1186/s12885-020-07421-z

**Published:** 2020-09-23

**Authors:** Hui-lei Miao, Ai-lin Zhao, Ming-hui Duan, Dao-bin Zhou, Xin-xin Cao, Jian Li

**Affiliations:** Department of Hematology, Peking Union Medical College Hospital, Chinese Academy of Medical Sciences and Peking Union Medical College, Beijing, 100730 People’s Republic of China

**Keywords:** Langerhans cell histiocytosis, Adult, Pulmonary involvement, Pulmonary function

## Abstract

**Background:**

The study aimed to investigate the clinical features and prognosis factors of adult patients with Langerhans cell histiocytosis (LCH) with pulmonary involvement, especially multisystem (MS) LCH with pulmonary involvement.

**Methods:**

We retrospectively analyzed the demographic materials, clinical features and treatment outcomes of 119 adult LCH patients with pulmonary involvement at our center from January 1990 to November 2019.

**Results:**

Among 119 patients, 13 (10.9%) had single-system (SS) LCH, and 106 (89.1%) had MS-LCH with pulmonary involvement. SS-LCH patients had higher smoking rate (84.6% vs 52.8%, *P* = 0.026) and smoking index (300 vs 200, *P* = 0.019) than MS-LCH patients. The percentage of respiratory symptoms of SS-LCH patients was higher than MS-LCH patients (84.6% vs 53.8%, *P* = 0.034). Pulmonary function was impaired in 83.8% of the patients, and DLCO was the parameter most frequently impaired, accounting for 81.1%. The median DLCO was 65.1% predicted. Patients with pneumothorax had significantly worse DLCO (*P* = 0.022), FEV1 (*P* = 0.000) and FEV1/FVC (*P* = 0.000) than those without pneumothorax. During the follow-up, 72.4% of the patients had stable pulmonary function, and 13.8% showed improvements after chemotherapy. The estimated 3-year OS and EFS were 89.7 and 58.3%, respectively. Patients with a baseline FEV1 ≤ 55% predicted had worse OS. A history of pneumothorax indicated worse EFS and cytarabine based therapy predicted better EFS.

**Conclusions:**

An FEV1 ≤ 55% predicted and a history of pneumothorax at diagnosis indicated a poor prognosis. Cytarabine based regimen may arrest the decline in pulmonary function in LCH patients with pulmonary involvement and improve EFS.

## Background

Langerhans cell histiocytosis (LCH) is a rare inflammatory myeloid neoplasm characterized by organ infiltration by pathological myeloid dendritic cells that share surface markers with epidermal Langerhans cells (CD1a+/CD207+) [1, 2]. LCH can develop in people of any age with different incidences. The incidence of LCH is approximately 5 to 9 per million in children and higher than 1 per million in adults [1]. LCH has heterogeneous clinical manifestations, ranging from single self-resolving lesions to life-threatening multiple organ damage. According to the number of organs involved, LCH is divided into single-system disease (SS) and multisystem disease (MS) [2]. As one of the most commonly affected organs, the lung can be involved as an isolated organ or as part of multisystemic LCH [3].

Pulmonary involvement in LCH, also known as PLCH, shows variable clinical courses and outcomes, ranging from asymptomatic processes to respiratory failure and death [4]. It has been reported that solitary pulmonary involvement is more common than MS-LCH in adults [4–7]. Nevertheless, the prevalence of PLCH is still unclear due to the nature of the disorder. As a consequence, previous studies have mostly described the features of solitary lung lesions, while pulmonary involvement in MS-LCH has been less described, thus limiting our understanding of the overall perspective of the disease. Furthermore, few studies have investigated the outcomes and prognosis of PLCH. The study conducted by the Histiocyte Society Adult Registry reported that LCH patients with isolated pulmonary lesions had much lower survival rates than those who had MS-LCH with lung involvement [8]. However, Delobbe et al. proposed that multi-organ involvement indicated poor prognosis [9]. The prognosis indicators are still controversial and unclear. As a result, describing the clinical features, treatment outcomes and prognoses of adult LCH patients with pulmonary involvement is necessary to help people understand this rare disease.

For this purpose, we retrospectively reviewed the medical records of adult patients with LCH with pulmonary involvement who were evaluated at our center over a 30-year period. We also analyzed the outcomes of these patients and identified risk factors that affect prognosis.

## Methods

### Patients

Patients who were diagnosed with LCH at Peking Union Medical College Hospital, China, between January 1990 and November 2019 were identified from our institutional database. The pathological diagnosis of LCH was confirmed by 2 experienced pathologists of Peking Union Medical College Hospital according to the World Health Organization classification of tumors [10]. The diagnosis of mixed histiocytosis (LCH & Erdheim-Chester Disease (ECD)) was based on previous criteria [11]. Pulmonary involvement was diagnosed based on one of the following criteria: 1) disease confirmed by lung biopsy; and 2) biopsy of other organs together with typical high-resolution computed tomographic (HRCT) findings [4] or positron emission tomography computed tomography (PET-CT) findings [12]. Risk organ included the liver, spleen and hematopoietic system and the involvement was defined based on the previous criteria [2, 13, 14]. Patients younger than 18 years of age at the time of the diagnosis were excluded. The study was performed in accordance with the ethical standards laid down in the 1964 Declaration of Helsinki and its later amendments. The study obtained waivers of informed consent and approval from the Peking Union Medical College Hospital Ethics Committee.

### Data collection

Clinical data including patient demographics, clinical presentation, smoking habits, coexisting medical conditions, the results of laboratory tests and HRCT, pulmonary function test (PFT), echocardiogram and arterial blood gas examinations, treatment regimens and outcomes were retrieved from the patients’ medical records. The pulmonary function data that were collected included total lung capacity (TLC), forced expiratory volume in 1 second (FEV1), the ratio of FEV1 to the forced vital capacity (FVC) determined by plethysmography, and the diffusion lung capacity for carbon monoxide (DLCO) determined by the single-breath method. Restrictive ventilatory dysfunction was defined as a TLC value that was less than 80% of the predicted value. Obstructive ventilatory dysfunction was defined as a ratio of FEV1/FVC less than 70%. Mixed ventilatory dysfunction was diagnosed if both of the criteria above were met. Diffusion dysfunction was defined as a DLCO value less than 80% [15]. The smoking index indicated each smoker’s cigarette consumption over a long period. The following equation was used to calculate smoking index = cigarettes smoked per day x years of cigarette use.

### Treatment and outcome

Systemic chemotherapy was divided into two types of regimen such as cytarabine based therapy and vindesine and prednisone (VP) based therapy. Cytarabine based therapy was further divided into methotrexate/cytarabine (MA) and cytarabine monotherapy. Concretely, the VP based regimen and MA regimen were administered according to the previous studies [16, 17]. Cytarabine 100 mg/m2 was administered subcutaneously or intravenously for 5 days every 35 days as monotherapy.

All patients were followed up by clinic records or by telephone. The last follow-up was December 15, 2019. Overall survival (OS) was defined as the time from diagnosis to death or the last follow-up. Event-free survival (EFS) was defined as the time from the initiation of systemic chemotherapy to the first event or the last follow up. Events were defined as a poor response to chemotherapy, reactivation after chemotherapy and death from any cause. Poor response referred to persistence of signs and symptoms, or progressive disease according to the current elevation criteria [18]. Patients without a recorded date of event were censored on the date of last contact. For the outcome of pulmonary function, improvement was defined as a percentage increase of more than 10% for FEV1 or FVC and 15% for DLCO, while deterioration was defined as reductions of the same percentages for the same parameters. If the changes were less than the defined percentage, lung function was considered stable [5, 19]. The overall pulmonary function outcome was defined based on increases or decreases of 10% in FEV1 and/or FVC and/or of 15% in DLCO. If improvement and deterioration coexisted, the impaired parameter was used as the overall pulmonary function outcome [5].

### Statistical analysis

Descriptive statistics were applied to present the demographic and some clinical characteristics of the patients. Categorical data are described as counts and proportions, and continuous data are described as medians and ranges. For categorical variables, the Chi-square test was used to compare the difference between groups, and Fisher’s exact test was used when the number of cases was < 5. The Mann–Whitney U test was used for continuous variables. Spearman rank correlation was used to explore the correlation between two variables. OS curves and EFS curves were plotted according to the Kaplan-Meier method, and the outcome differences between groups were estimated by log-rank tests. Risk factors were investigated using Cox regression models. Receiver operating characteristic curve with death was used to identify the threshold for FEV1 and the number of involved organs, which were then analyzed as dichotomous variables. Hazard ratios (HRs) and their 95% confidence intervals (CIs) were calculated using multivariate Cox proportional hazards regression models. Statistical analysis was performed using SPSS software (v23.0; IBM, Armonk, NY, USA). All statistical tests were two-sided, and *P* < 0.05 was considered to be statistically significant, while candidate variables with a *P* value < 0.1 in the univariate analysis were included in the multivariable model.

## Results

### Patients

There were 237 adult LCH patients diagnosed in Peking Union Medical College Hospital between January 1990 and November 2019 in total. Of them, 119 (50.2%) patients with pulmonary involvement were included in this study.

The baseline demographics and clinical characteristics are summarized in Table 1. Of the 119 patients, the median age at diagnosis was 33 years (range: 18–64 years). Eighty-seven (73.1%) patients were male, with a male to female ratio of 2.72. The median time from symptom onset to diagnosis of the disease was 17 months (range: 1–268 months). One (0.8%) patient was diagnosed before 2000, 19 patients (16.0%) were diagnosed from 2000 to 2009, and 99 patients (83.2%) were diagnosed from 2010 to 2019 (supplementary Fig. 1). In addition, 6 patients had mixed histiocytosis (LCH & ECD). Among all the patients, 13 (10.9%) patients had biopsy-confirmed SS-LCH, which meant isolated pulmonary LCH, and 106 (89.1%) had MS-LCH with pulmonary involvement. There were no significant differences in age, sex, or time to diagnosis between patients with SS-LCH and those with MS-LCH. The median number of organs involved was 3 (range: 1–8) among patients with MS-LCH. Among MS-LCH patients, 31 (29.2%) patients had liver involvement, 11 (10.4%) had spleen involvement and 8 (7.5%) patients had the combination of liver and spleen involvement. However, no patient had hematopoietic involvement in our cohort. Consequently, a total of 34 (32.1%) patients had risk organ involvement.

**Table 1:** The demographics and clinical characteristics of the patients at the time of diagnosis

Characteristics	All patients *N* = 119
Age, years (median, range)	33 (18–64)
Male, n (%)	87 (73.1)
Time to diagnosis, months (median, range)	17 (1–268)
Smoking status	*N* = 111
Smokers, n (%)	67 (60.4)
Non-smokers, n (%)	44 (39.6)
Smoking index of smokers (median, range)	200 (2–1600)
Organ involvement	
SS-LCH, n (%)	13 (10.9)
MS-LCH, n (%)	106 (89.1)

*SS* Single-system disease, *MS* Multisystem disease

A total of 111 patients had data regarding their smoking habits, including 67 (60.4%) patients who were current smokers or ex-smokers, and their median smoking index was 200 (range: 2–1600). SS-LCH patients had higher smoking rate (84.6% vs 52.8%, *P* = 0.026) and median smoking index (300 vs 200, *P* = 0.019) than MS-LCH patients. Furthermore, among the 119 patients, 3 patients were diagnosed with other malignant neoplasms. One patient suffered from giant cell tumor of the tibia before the onset of LCH. Another patient was diagnosed T-cell lymphoma before the diagnosis but after the onset of LCH, and one patient was diagnosed follicular lymphoma 4 years after the diagnosis of LCH.

### Symptoms and organ involvement

The clinical manifestations of the 119 patients are shown in Table 2, and the data are divided into SS-LCH and MS-LCH with pulmonary involvement. Among the 13 patients with isolated pulmonary involvement, 2 patients had no symptoms, and 11 patients had respiratory symptoms (84.6%), including cough (*n* = 9, 69.2%), exertional dyspnea (n = 9, 69.2%) and spontaneous pneumothorax (*n* = 5, 38.5%). One patient had bilateral pneumothorax. Of the 106 MS-LCH patients, one had no symptoms, and 57 patients had respiratory symptoms (53.8%) and 98 patients had extrapulmonary symptoms (92.5%). Respiratory manifestations in the MS group included cough (*n* = 33, 31.1%), exertional dyspnea (*n* = 24, 22.6%), spontaneous pneumothorax (*n* = 25, 23.6%) and hemoptysis (*n* = 4,3.8%). Ten patients suffered from recurrent bilateral pneumothorax (9.4%). The percentage of respiratory symptoms in MS-LCH patients was significantly lower than that in SS-LCH patients (*P* = 0.034). The percentage of cough (*P* = 0.012) and exertional dyspnea (*P* = 0.001) in MS-LCH patients was lower than that in SS-LCH patients, while the percentage of pneumothorax showed no significant difference (*P* = 0.309). Extrapulmonary symptoms were associated with lesion location and mainly included diabetes insipidus, bone pain, rash, lymph node enlargement, and suppurative otitis media. Additionally, patients with no symptoms were identified by imaging findings and diagnosed by lung biopsy.

**Table 2:** The symptoms of patients at diagnosis

	SS-LCH (*N* = 13)	MS-LCH (*N* = 106)	*P* value
No symptom, n (%)	2 (15.4)	1 (0.9)	0.031
Respiratory symptoms, n (%)	11 (84.6)	57 (53.8)	0.034
Cough, n (%)	9 (69.2)	33 (31.1)	0.012
Exertional dyspnea, n (%)	9 (69.2)	24 (22.6)	0.001
Pneumothorax, n (%)	5 (28.5)	25 (23.6)	0.309
Hemoptysis, n (%)	0 (0)	4 (3.8)	
Extra pulmonary symptoms, n (%)	0 (0)	98 (92.5)	

### Radiological features

**Fig. 1:**
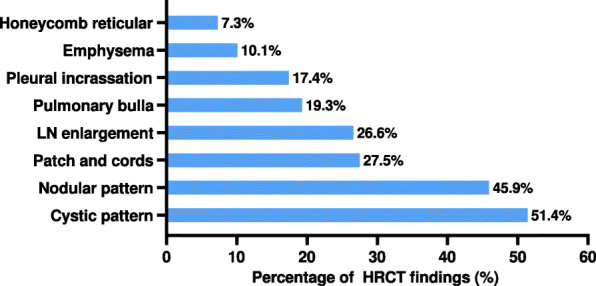
HRCT findings of LCH patients with pulmonary involvement at diagnosis LN, lymph node.

**Fig. 2:**
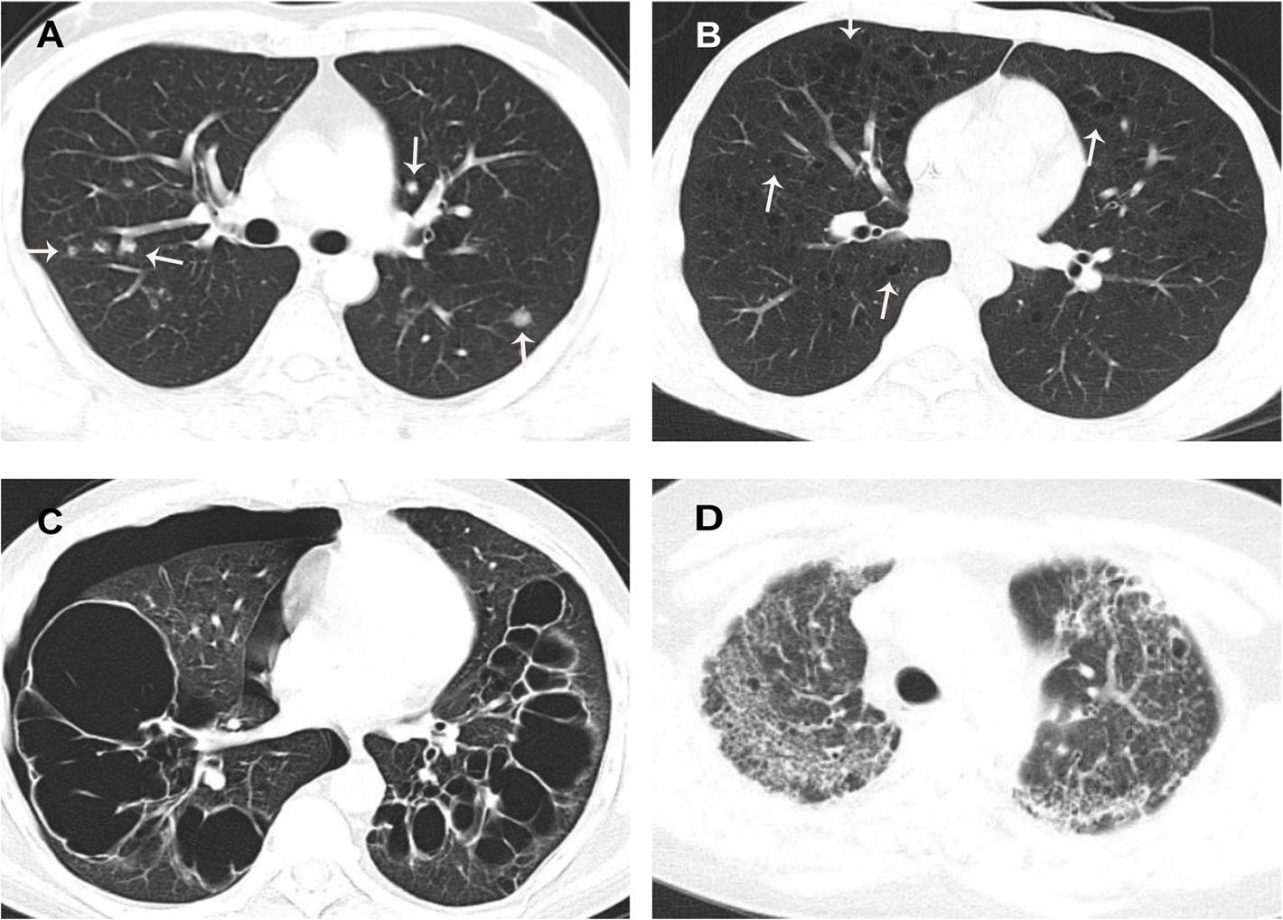
HRCT imaging findings of adult LCH patients with pulmonary involvement: (**a**) multiple modules (white arrows), (**b**) thin-walled cysts (white arrows) and emphysema, (**c**) pulmonary bulla, and (**d**) honeycomb and reticular patterns

A total of 109 of 119 patients underwent HRCT examinations. HRCT findings showed diversity (Fig. 1), which mainly manifested as interstitial lung disease changes. The most common HRCT findings are shown in Fig. 2 and were cystic patterns (*n* = 56, 51.4%), followed by nodular patterns (*n* = 50, 45.9%), patch and cord shadows (*n* = 30, 27.5%), multiple lymph node (LN) enlargement (*n* = 29, 26.6%), pulmonary bulla (*n* = 21,19.3%), pleural incrassation (*n* = 19,17.4%), emphysema (*n* = 11,10.1%) and honeycomb and reticular patterns (*n* = 8, 7.3%). Twenty-five patients (22.9%) had both nodular and cystic features. Furthermore, multiple enlarged lymph nodes mainly referred to the mediastinal (*n* = 25, 22.9%), axillary (*n* = 9, 8.3%) and hilar (n = 9, 8.3%) lymph nodes.

In total, 50 of 119 patients underwent echocardiogram examinations. One patient showed severe pulmonary arterial hypertension (PAH), a pulmonary arterial systolic pressure (PASP) of 95 mmHg, right heart enlargement, inferior vena cava widening and severe tricuspid regurgitation, combined with slight pericardial effusion. One patient had mild right ventricular hypertrophy, with a PASP of 42 mmHg. Four patients had left atrial enlargement, and 2 of them had mild mitral valve insufficiency. The remaining 44 patients presented near normal results.

### Pulmonary function findings and arterial blood gas results

Seventy-four patients had complete PFT data (Table 3). Twelve (16.2%) patients had normal pulmonary function, and 62 (83.8%) patients had ventilatory and/or diffusion dysfunction. A restrictive pattern was found in 19 (25.7%) patients, and an obstructive pattern was found in 12 (16.2%) patients. Two (2.7%) patients showed mixed ventilation disorder and 60 patients (81.1%) showed diffusion disorder. Among the patients with defective diffusing capacity, 33 (44.6%) patients had isolated diffusing capacity defects with normal ventilatory function. Sixteen (21.6%) patients had restrictive defects, and 9 (12.2%) had obstructive defects. Two patients had diffusing capacity defects and mixed ventilation disorders. The median percentage of the predicted DLCO value was 65.1% (range: 27.6–119%), while the median values of FEV1, FEV1/FVC and TLC were normal. However, the severity of both ventilatory and diffusion defects were extremely different among patients, ranging from minor abnormalities to very severe damage. The results for the whole dataset are shown in Table 3. Among the 74 patients, patients with a history of pneumothorax had a significantly worse DLCO (52.2% vs 66.4%, *P* = 0.022), FEV1 (46.1% vs 87.0%, *P* = 0.000) and FEV1/FVC (74.1% vs 80.9%, *P* = 0.000) than patients without pneumothorax.

**Table 3:** Pulmonary function parameters of LCH patients with pulmonary involvement at diagnosis

Pulmonary function test	*N* = 74
Normal, n (%)	12 (16.2)
Restrictive disorders, n (%)	19 (25.7)
Obstructive disorders, n (%)	12 (16.2)
Defective diffusing capacity, n (%)	60 (81.1)
Isolated diffusing capacity defect, n (%)	33 (44.6)
Restrictive and diffusing capacity defect, n (%)	16 (21.6)
Obstructive and diffusing capacity defect, n (%)	9 (12.2)
Mixed ventilation and diffusing capacity defect, n(%)	2 (2.7)
DLCO, % of predicted (median, range)	65.1 (27.6–119.0)
FEV1, % of predicted (median, range)	82.7 (26–109.8)
FEV1/FVC, % (median, range)	79.90 (50.75–106.0)
TLC, % of predicted (median, range)	87.4 (51.9–188.1)

Forty-two patients underwent arterial blood gas analysis. The median arterial partial pressure of oxygen (PaO_2_) was 81.35 mmHg (range: 43–110 mmHg) under room air. The PaO_2_ of 19 patients was lower than 80 mmHg, including 3 patients with type I respiratory failure. Two of the 3 patients died from the disease, and one patient had disease reactivation but was still alive at the last follow-up.

### Evolution of PFT during follow-up

During follow-up, 29 patients were re-evaluated with PFTs after treatment, and all of them had MS-LCH and received MA regimen as systemic chemotherapy. Table 4 shows the pulmonary function outcomes of these patients. According to the criteria mentioned above, the pulmonary function of 21 (72.4%) patients was stable after treatment. Two patients had deteriorated DLCO. The predicted DLCO of one patient decreased from 64.7 to 43.7%, and the other decreased from 87.7 to 53.6%. One patient had deteriorated FEV1 values, from 66.5 to 47.3%. One patient had TLC deterioration which decreased from 79.6 to 69%. Two patients showed improvements in DLCO. One increased from 74.6 to 91.6% and the other patient increased from 46.7 to 67.7%. Four patients had improved FEV1 values, which increased from 75.6 to 87%, from 75.9 to 95%, from 75.4 to 95.4% and from 47.7 to 64.9%. One patient showed improvements in TLC, which increased from 77.2 to 89%. A total of 4 (13.8%) patients achieved pulmonary function improvements in at least one parameter, and 4 (13.8%) patients had deterioration of at least one pulmonary function parameter as well.

**Table 4:** Pulmonary function outcomes of 29 patients

PFT, n (%)	Improvement	Deterioration	Stabilization
DLCO	2 (6.9)	2 (6.9)	25 (86.2)
FEV1	4 (13.8)	1 (3.4)	24 (82.8)
TLC	1 (3.4)	1 (3.4)	27 (93.1)
Overall	4 (13.8)	4 (13.8)	21 (72.4)

### Treatment and survival outcomes

The treatment options of 119 patients were listed in supplementary Fig. 2. At the time of diagnosis, all current smokers were advised to stop smoking. In 13 LCH patients with isolated pulmonary involvement, one patient received VP based chemotherapy. One patient had severe respiratory failure and was recommended to undergo lung transplantation but died within 3 months before surgical intervention. The remaining 11 patients were advised to undergo observation. Three of these patients were lost to follow-up, and 8 patients were in stable condition at the last follow-up. Among the 106 MS-LCH patients with pulmonary involvement, 89 patients received systemic chemotherapy and 5 patients were treated with prednisone alone. Of the 89 patients, 65 received cytarabine based regimen, included that 59 patients received MA regimen and 6 patients received cytarabine monotherapy. Twenty-four patients received VP based chemotherapy. Twelve patients did not receive therapy for reasons including personal willingness, visits to other hospitals or poor condition unsuitable for chemotherapy.

**Fig. 3:**
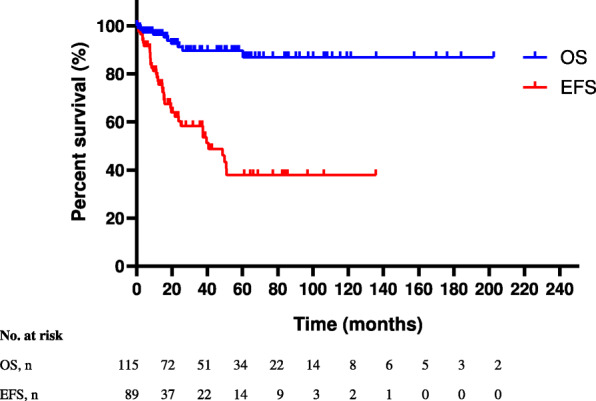
Overall survival (OS) of the 119 patients and event-free survival (EFS) of 89 patients who received systemic chemotherapy. *# Four patients were lost to follow-up once the diagnosis was made*

The median follow-up time for the whole cohort was 33 months (range: 1–203 months). The 3-year estimated OS was 89.7%. Ten patients died. Of them, one was an SS-LCH patient and 9 were MS-LCH patients. One patient died from VP-based therapy related infection and the other 9 patients died from disease progression, of whom 4 patients died from pulmonary dysfunction and 5 patients died from the combination of pulmonary and liver dysfunction. There was no significant difference between the SS-LCH and MS-LCH patients in OS (*P* = 0.874). Of the 89 MS-LCH patients who received systemic chemotherapy, 30 patients had disease reactivation after chemotherapy, and 6 patients had poor responses to chemotherapy and one patient died from VP-based therapy related infection. The median EFS was 40.8 months (95% CI, 19.0–62.5 months), and the 3-year estimated EFS was 58.3%. The OS and EFS were shown in Fig. 3.

Univariate analysis was conducted to explore the prognostic factors of OS and EFS and incorporated age at diagnosis, sex, smoking status, the number of involved organs, pneumothorax, DLCO, FEV1, TLC, risk organ involvement and treatment in 89 MS-LCH patients who received systemic chemotherapy. Then, age at diagnosis, FEV1, TLC and cytarabine based treatment were included in the multivariate analysis of OS, and FEV1 ≤ 55% independently indicated a worse OS (3-year estimated OS: 78.5% vs 98.3%, *P* = 0.028) (Table 5). Multivariate Cox regression of EFS was conducted using smoking status, number of involved organs, pneumothorax, spleen involvement and cytarabine based therapy. Patients with pneumothorax had a worse EFS (19.5 months vs 50.8 months, *P* = 0.046), while patients who received cytarabine based therapy had a better EFS (40.8 months vs 12.0 months, *P* = 0.065) (Table 6).

**Table 5:** Prognostic factors for overall survival of 89 MS-LCH patients who received systemic chemotherapy through univariate and multivariate cox regression *Only factors with a *P* value < 0.1 in univariate analysis were listed

	Univariate Cox		Multivariate Cox	
	HR (95% CI)	*P* value	HR (95% CI)	*P* value
Age at diagnosis	1.080 (1.010–1.155)	0.024	1.051 (0.923–1.197)	0.453
FEV1 ≤ 55%	14.882 (1.339–164.037)	0.028	12.487 (1.131–137.822)	0.039
TLC	0.907 (0.832–0.988)	0.025	0.864 (0.703–1.061)	0.162
Cytarabine based therapy	0.127 (0.024–0.662)	0.014	0.151 (0.009–2.456)	0.184

*MS* Multisystem disease

**Table 6:** Prognostic factors for event-free survival of 89 MS-LCH patients who received systemic chemotherapy through univariate and multivariate cox regression *Only factors with a *P* value < 0.1 in univariate analysis were listed

	Univariate Cox		Multivariate Cox	
	HR (95% CI)	*P* value	HR (95% CI)	*P* value
Smoking status	0.517 (0.257–1.042)	0.065	0.553 (0.269–1.141)	0.109
More than 3 organs involved	1.818 (0.920–3592)	0.080	1.647 (0.718–3.777)	0.239
Pneumothorax	1.989 (1.012–3.910)	0.046	3.203 (1.454–7.053)	0.004
Spleen	2.159 (0.884–5.276)	0.091	1.630 (0.605–4.394)	0.334
Cytarabine based therapy	0.525 (0.262–1.053)	0.065	0.325 (0.141–0.749)	0.008

*MS* Multisystem disease

## Discussion

This is one of the largest cohort studies to describe the clinical manifestations, examination features, treatment and outcomes and explore the risk factors of adult LCH patients with pulmonary involvement. Our study extended our understanding of this rare disease in adults, especially MS-LCH with pulmonary involvement.

In our 119 patients, 13 had isolated pulmonary involvement, and 106 had MS-LCH with pulmonary involvement. Previous studies on PLCH have mainly concentrated on isolated pulmonary LCH [6, 7], and studies on MS-LCH have focused more on systemic manifestations and management [2, 8], while detailed descriptions and evaluations of pulmonary involvement features in MS-LCH patients are rare. Our study showed that the smoking rate and index were significantly higher in patients with isolated pulmonary involvement than in those with MS-LCH. The specific numbers in the two groups were consistent with previous studies on SS-LCH [4] and MS-LCH [8]. In addition, SS-LCH patients were more likely to present respiratory symptoms, including cough and exertional dyspnea. The proportion of respiratory symptoms in SS-LCH patients was similar to that in a previous study [3].

Among our 74 patients with available PFT results, DLCO was the most affected PFT parameter. The diffusing capacity could be impaired by itself or accompanied by restrictive, obstructive or mixed ventilation abnormalities, which was in agreement with previous studies [4, 7]. Radzikowska et al. presented that patients with pneumothorax had worse FEV, FEV1 and TLC than those without pneumothorax in a cohort involving 90 patients [20]. We also found that patients with a history of pneumothorax had worse DLCO, FEV1 and FEV1/FVC. Therefore, patients who had a history of pneumothorax at the time of diagnosis had greater respiratory impairment than patients without pneumothorax, and the most relevant parameter still needs to be verified.

A multicenter and prospective study demonstrated that a substantial proportion of PLCH patients suffered pulmonary function deterioration within 2 years [21]. Grobost et al. demonstrated that cladribine chemotherapy kept stable or slightly improved pulmonary function in all 5 cases [22]. We also tracked the improvements or deterioration in pulmonary function of 29 patients who received systemic cytarabine based regimen. Of these patients, 72.4% had stable pulmonary function, and 13.8% had improvements. As a consequence, cytarabine based regimen may arrest the decline of pulmonary function rather than obviously improve pulmonary function. The protection of pulmonary function in PLCH patients still depends on early diagnosis and early treatment to arrest deterioration.

The study from the Histiocyte Society Adult Registry presented that patients with isolated pulmonary involvement had a higher mortality rate than those with MS-LCH [8], while Basset’s study proposed that multiple organ involvement suggested adverse outcomes [23]. Vassallo’s study also proposed that pulmonary function could probably be a prognostic factor [4]. These data were confounded by many factors, such as uncertainty in the diagnosis and the lack of a multivariate analysis; thus, no general conclusion was drawn regarding the prognostic factors of adult PLCH patients. We demonstrated that a percentage of the predicted FEV1 ≤ 55% indicated worse survival. A history of pneumothorax at diagnosis independently related to worse EFS. Risk organ involvement indicated poor prognosis in children [2, 13, 14]. Cao et al. firstly reported the involvement of liver predicted a worse prognosis in adult LCH patients [17]. In our cohort, neither liver nor spleen involvement was associated with poor prognosis. This was probably because we only enrolled the adult LCH patients with pulmonary involvement. More large-scale studies are needed to investigate it.

There are several limitations in our study. Our study is a single-center retrospective study and had its intrinsic limitations. For example, the PFT was not uniformly or prospectively followed-up and sometimes based on the physicians’ judgements, which might cause bias in the assessment of the PFT changes. The incomplete data also made it difficult to evaluate the effect of chemotherapy on PFT changes in the whole cohort. In addition, our cohort lacked molecular data including *BRAF*^*V600E*^ mutations and other somatic mutations, however the current study primarily focused on clinical characteristics and prognostic analysis. In the future, more prospective studies should be conducted to investigate it.

## Conclusions

Our study found that a baseline FEV1 ≤ 55% predicted and a history of pneumothorax at diagnosis indicated a worse prognosis in MS-LCH patients with pulmonary involvement. Cytarabine based regimen may arrest the decline in pulmonary function of MS-LCH patients with pulmonary involvement rather than obviously improve pulmonary function.

## Supplementary information

**Additional file 1.**

## Data Availability

The datasets used and/or analysed during the current study are available from the corresponding author on reasonable request.

## Abbreviations

DLCO: Diffusion lung capacity for carbon monoxide
FEV1: Forced expiratory volume in one second
FVC: Forced vital capacity
HRCT: High-resolution computed tomographic
LCH: Langerhans cell histiocytosis
MS-LCH: Multisystem Langerhans cell histiocytosis
PaO_2_: Partial pressure of oxygen in the blood
PASP: Pulmonary arterial systolic pressure
PET-CT: Positron emission tomography computed tomography
PFT: Pulmonary function test
PLCH: Pulmonary Langerhans cell histiocytosis
SS-LCH: Single-system Langerhans cell histiocytosis
TLC: Total lung capacity
