# Supplementary material for: Clinical presentation and prognostic analysis of adult patients with Langerhans cell histiocytosis with pulmonary involvement
Source: BMC Cancer. 2020 Sep 23;20:911. doi: 10.1186/s12885-020-07421-z (PMC7513534; doi:10.1186/s12885-020-07421-z)
Supplement: Supplementary file 1 — Additional file 1. [file 12885_2020_7421_MOESM1_ESM.docx]

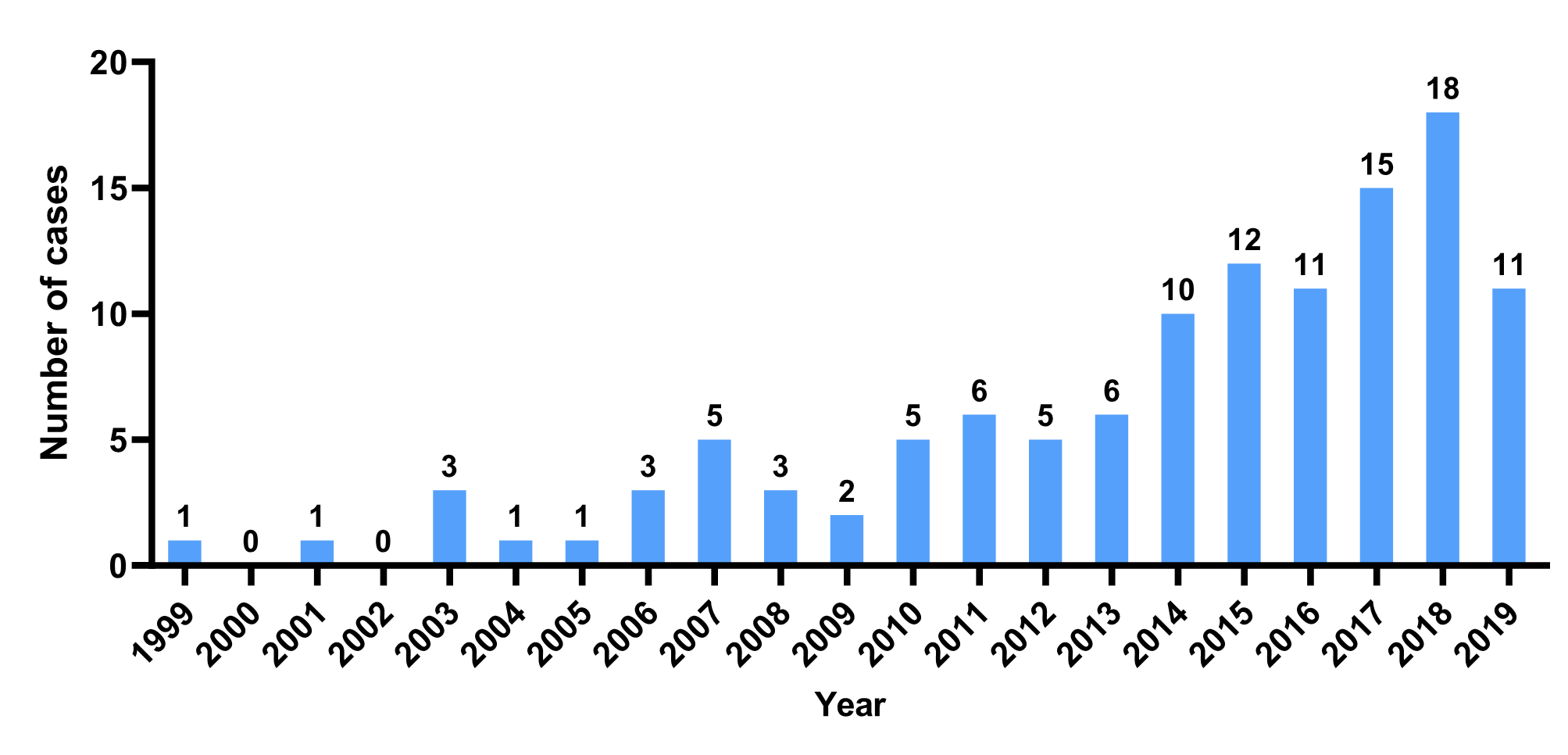


**Supplementary Fig. 1** Year of diagnosis of of LCH with pulmonary involvement in 119 patients


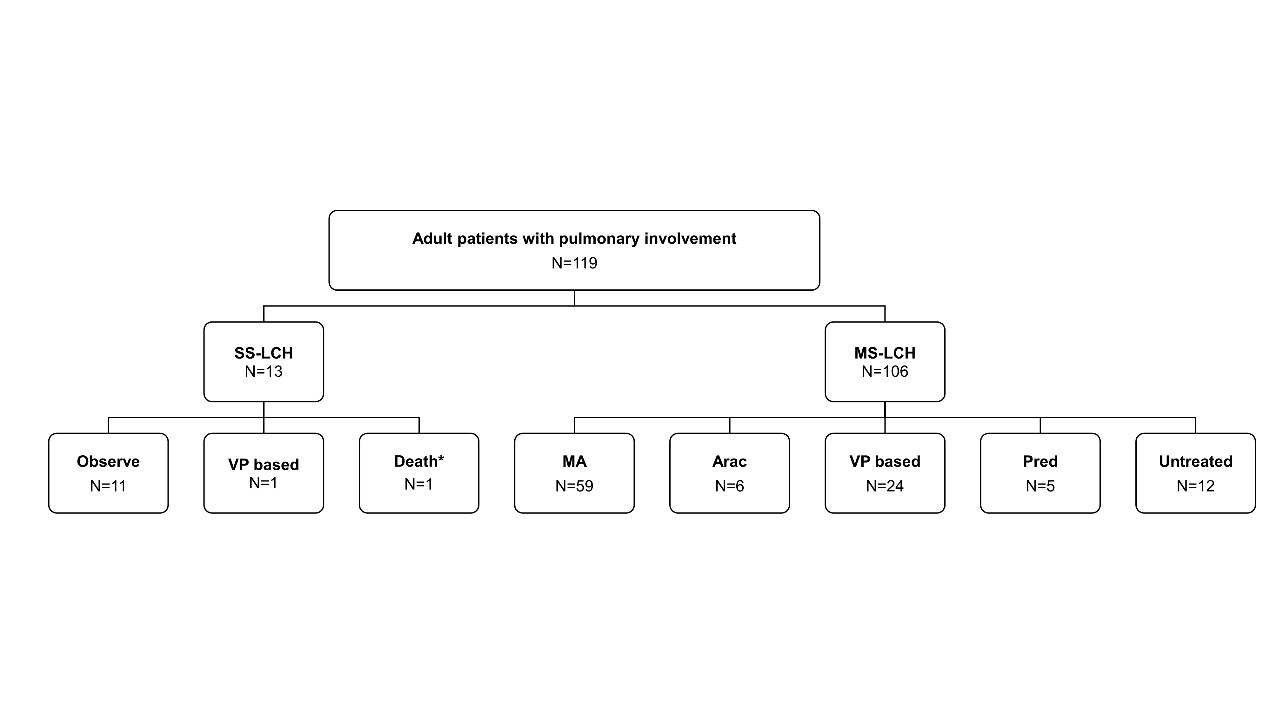


**Supplementary Fig. 2** Treatment of 119 adults patients with pulmonary involvement

*SS, single-system disease; MS, multisystem disease; VP, vindesine and prednisone-based therapy; MA, methotrexate and cytarabine; Arac, cytarabine; pred, prednisone*

**patient with severe respiratory failure, died before lung transplantation*
